# The In Vivo and In Vitro Toxicokinetics of Citreoviridin Extracted from *Penicillium citreonigrum*

**DOI:** 10.3390/toxins11060360

**Published:** 2019-06-20

**Authors:** Yosuke Uchiyama, Masahiko Takino, Michiko Noguchi, Nozomi Shiratori, Naoki Kobayashi, Yoshiko Sugita-Konishi

**Affiliations:** 1 The Graduate School of Life and Environmental Sciences, Department of Food and Life Sciences, Azabu University, 1-17-71 Fuchinobe, Chuo-ku, Sagamihara-shi, Kanagawa 252-5201, Japan; de1701@azabu-u.ac.jp (Y.U.); shiratorin@aqua-ckc.co.jp (N.S.); n-kobayashi@azabu-u.ac.jp (N.K.); 2 Agilent Technologies, Japan, Ltd., 9-1 Takakura-cho, Hachioji, Tokyo 192-8510, Japan; masahiko_takino@agilent.com; 3 Laboratory of Theriogenology, Department of Veterinary Medicine, Azabu University, 1-17-71 Fuchinobe, Chuo-ku, Sagamihara-shi, Kanagawa 252-5201, Japan; m-noguchi@azabu-u.ac.jp

**Keywords:** citreoviridin, toxicokinetics, bioavailability, swine, Caco-2, S9

## Abstract

Citreoviridin (CTVD), a mycotoxin called yellow rice toxin, is reported to be related to acute cardiac beriberi; however, its toxicokinetics remain unclear. The present study elucidated the toxicokinetics through in vivo experiments in swine and predicted the human toxicokinetics by comparing the findings to those from in vitro experiments. In vivo experiments revealed the high bioavailability of CTVD (116.4%) in swine. An intestinal permeability study using Caco-2 cells to estimate the toxicokinetics in humans showed that CTVD has a high permeability coefficient. When CTVD was incubated with hepatic S9 fraction from swine and humans, hydroxylation and methylation, desaturation, and dihydroxylation derivatives were produced as the predominant metabolites. The levels of these products produced using human S9 were higher than those obtained swine S9, while CTVD glucuronide was produced slowly in human S9 in comparison to swine S9. Furthermore, the elimination of CTVD by human S9 was significantly more rapid in comparison to that by swine S9. These results suggest that CTVD is easily absorbed in swine and that it remains in the body where it is slowly metabolized. In contrast, the absorption of CTVD in humans would be the same as that in swine, although its elimination would be faster.

## 1. Introduction

**Figure 1:**
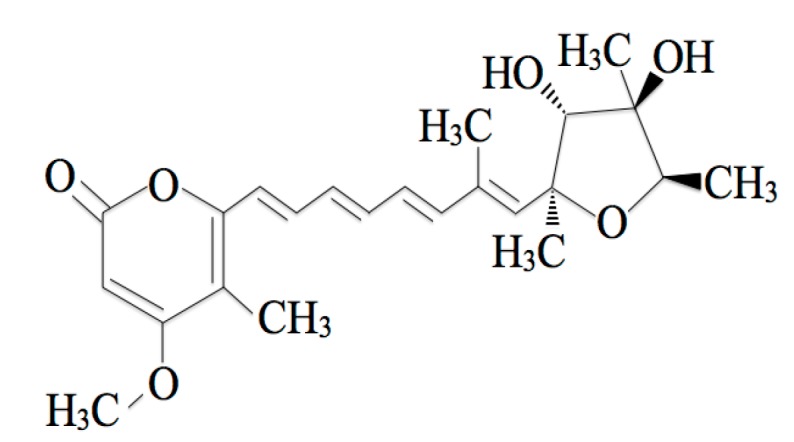
The chemical structure of citreoviridin (CTVD).

Citreoviridin (CTVD) (Figure 1) is a mycotoxin produced as a secondary metabolite of *Penicillium citreonigrum*, *Aspergillus terreus*, and *Eupenicillium ochrosalmoneum* [1]. CTVD has previously been detected in food including rice in various areas [2,3,4,5,6]. Contamination with CTVD, well-known as yellow rice toxin, often occurs due to inappropriate management after harvest [7]. The toxicity of CTVD is mainly accompanied by ascending paralysis, central nervous system disturbance and respiratory arrest, and fatal adverse effects may occur [8]. Because CTVD is mainly found as a contaminant in rice, these properties of CTVD can be a serious problem in countries in which people consume rice as a staple food such as Thailand, Brazil, and Japan.

In a 2006 outbreak of beriberi in Brazil, 1207 people were diagnosed with beriberi, and 40 patients died [9]. Since *P. citreonigrum* and CTVD were detected in rice samples, CTVD was the suspected cause of the beriberi outbreak in that area [10].

Regarding the relationship between CTVD and beriberi, Uraguchi et al. reported, based on the results of an epidemiological investigation, the possibility of acute cardiac beriberi, which induces neurological symptoms and heart failure [11]. Ueno et al. reported that CTVD produced neurological symptoms similar to acute cardiac beriberi in humans based on experiments in mice and rats [12]. This disease has been caused by the consumption of moldy rice in areas in which rice preservation was inadequate. Studies on CTVD toxicity were performed using animal experiments from the 1940s to 1980s. These studies revealed that CTVD causes fatal adverse effects, with symptoms by ascending paralysis, disturbance of the central nervous system, and respiratory arrest [8]. The lethal dose 50% (LD_50_) of CTVD in mice was reported to be 3.6–11.8 mg/kg subcutaneously and 7.5 mg/kg intraperitoneally [13,14]. When crude extract from yellow rice was administered to several mammals subcutaneously, intraperitoneally, and per os, the abovementioned typical neurological symptoms were observed [15]. In addition, the development of these symptoms in rats occurred earlier when the dose was increased [16]. Chronic exposure to CTVD has also been reported as a possible trigger of Keshan disease (an endemic disease in which patients present cardiomyopathy) [17]. Sakai et al. focused on the chronic toxicity at low doses, and reported that rats died when 1/300 of the oral LD_50_ dose was administered subcutaneously every day for a six-month period [16]. This suggested that CTVD might accumulate in the body and have cumulative effects. Furthermore, the distribution of CTVD in the liver, kidney, heart, and brain, as determined by a photometric analysis, showed that CTVD remained in the liver, kidney, and heart of rats even at 52 h after its subcutaneous administration (SC) [12]. Although these results show the possibility that CTVD may easily remain in the body, the kinetics parameters, including oral bioavailability, have rarely been described.

The present study considered the cumulative properties of CTVD by elucidating the toxicokinetics and bioavailability of CTVD in vivo. Moreover, to estimate the bioavailability of CTVD in humans, the metabolic efficiency of CTVD in vitro was compared by an S9 metabolic study, and a permeability study was conducted using Caco-2 cells as a human intestinal cell model. First, the kinetics of CTVD in plasma were investigated by administering CTVD intravenously and orally to swine in an in vivo study. Subsequently, the intestinal permeability of CTVD was evaluated using Caco-2 cells and the metabolism of CTVD and the production of its main metabolites were investigated using S9 fractions obtained from swine and humans. The parameters thus obtained may contribute to improving the knowledge on the risk of CTVD.

## 2. Results

### 2.1. The Toxicokinetics of CTVD in Swine

**Figure 2:**
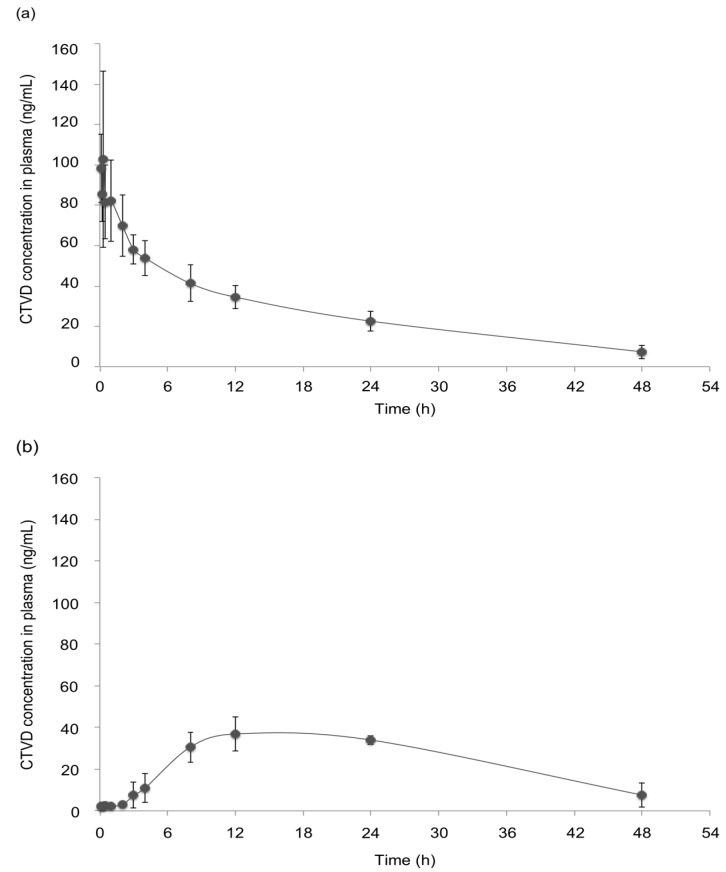
The CTVD concentration–time profiles in the plasma of swine after IV and oral administration (PO). CTVD (0.1 mg/kg·BW) was administered intravenously (**a**) and orally (**b**). Control plasma was obtained on the day before administration. Values are presented as the mean ± standard deviation (SD). *n* = 4 in both groups.

None of the swine that received CTVD intravenously or orally showed adverse clinical signs. The plasma CTVD concentration profile after intravenous administration (IV) is shown in Figure 2a. The rate constant (Kel) was small (0.5 ± 0.1 × 10^−1^ h^−1^) (Table 1). The mean half-life of CTVD was 16.2 ± 4.3 h, which was relatively long. The volume of distribution (Vd) was greater than the total body water (1.5 ± 0.2 L), and the mean residence time (MRT) of CTVD was estimated to be 14.6 ± 1.2 h. To calculate the bioavailability of CTVD based on the different estimated parameters, the area under the curve (AUC) was determined to be 1512.9 ± 331.7 h·ng/mL, with extrapolation to infinity.

The CTVD concentration profile in plasma after PO administration is shown in Figure 2b. The peak plasma concentration (C_max_, 38.2 ± 6.7 ng/mL) was observed at 15.0 ± 6.0 h (T_max_) after PO (Table 1). The Kel was 0.4 ± 0.2 ×10^−1^ h^−1^, which was low and similar to the value after IV administration. The T_1/2_ was approximately one day (21.4 ± 12.7 h), and the Vd/F was relatively large (1.7 ± 0.3 L). The MRT obtained from those values was relatively long (19.6 ± 4.0 h) (Table 1), suggesting that CTVD persisted in the bodies of swine. The AUC extrapolated to infinity was 1761.1 ± 813.5 h·ng/mL. The estimated bioavailabilities in swine from AUC_t_ and AUC∞ was 79.3% and 116.4%, respectively.

### 2.2. Permeability Study Using Caco-2 Cells

The results of the administration study showed that CTVD had high bioavailability in swine. In order to compare the intestinal permeability of CTVD in humans with the bioavailability of CTVD in swine, the permeability was investigated using Caco-2 cells. The Caco-2 cell model is an in vitro model used to evaluate intestinal permeability and the influence of chemical compounds on the intestinal barrier function in humans.

The apparent permeability coefficient (Papp) estimated from a Caco-2 permeability assay has been reported to be well correlated with the human in vivo absorption data for many agents [18,19]. The transepithelial electrical resistance (TEER) value is generally accepted to reflect the integrity of the tight junction dynamics in Caco-2 cells [20]; thus, the TEER was measured at 1 and 2 h after exposure to 3 and 10 µmol/L of CTVD. Our results showed no marked change in the TEER over time at any concentration (data not shown). The rate of CTVD transport from the apical (AP) side to the basolateral (BL) side was calculated based on the concentration of CTVD in the BL compartment. The Papp was calculated as described in a previous paper [21]. The Papp after 2 h of incubation with 3 and 10 µmol/L CTVD was 52.2 × 10^−6^ and 42.6 × 10^−6^ (cm/s), respectively, in the AP-BL direction (Table 2). These findings indicated that human intestinal cells were highly permeable to CTVD in vitro.

### 2.3. CTVD Elimination and the CTVD Metabolite Profile Following Incubation with S9 Fraction In Vitro

The fact that it took more than 40 h to eliminate CTVD in plasma after IV or PO administration in swine in vivo experiments suggested that CTVD was poorly metabolized in the liver. Thus, to confirm the metabolic activity of CTVD and the metabolites of CTVD produced in the liver, the residual CTVD concentration was measured after incubation in vitro with hepatic S9 fraction from swine, and the profile of the CTVD metabolites produced was determined using Q-TOF. These data obtained from the swine S9 fraction were subsequently compared to those obtained using human S9 fraction.

**Figure 3:**
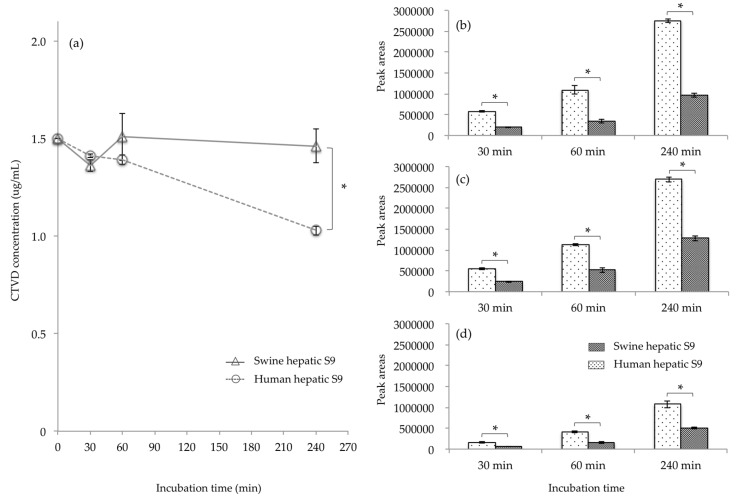
The comparison of the CTVD concentration and the main metabolites in humans and swine, obtained by incubating of CTVD with hepatic S9 fractions of humans and swine. CTVD (1.5 µg/mL) was incubated with the hepatic S9 fractions of humans and swine supplemented with NADP as a coenzyme. The CTVD concentrations at 30, 60, and 240 min after the start of incubation are described (**a**). The main metabolites produced in humans and swine are shown in (**b**–**d**). The metabolites in humans and swine at each time-point were compared based on the mean peak area of each metabolite. (**b**–**d**) show the mean peak areas of hydroxylation and methylation, desaturation, and dihydroxylation derivatives, respectively, following incubation with human or swine hepatic S9, respectively. Values are presented as the mean ± SD. Asterisks indicate a statistically significant difference (*p* < 0.05).

**Figure A1:**
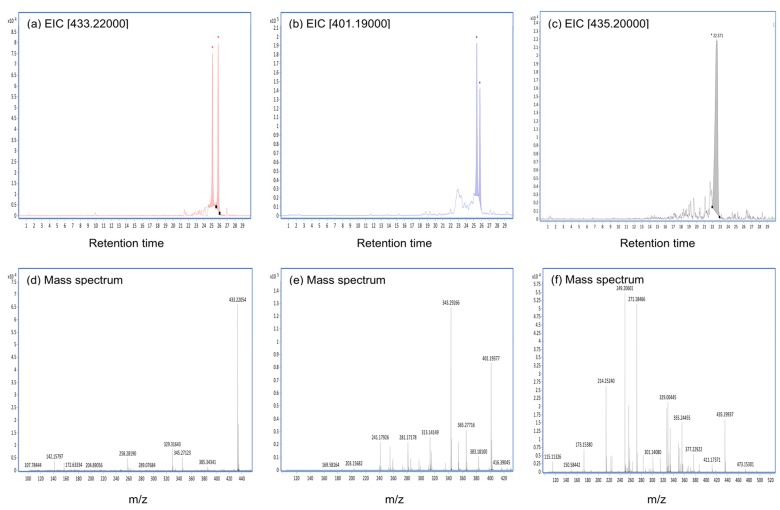
The extracted ion chromatogram (EIC) and mass spectra of the main metabolites of CTVD incubated with S9. The EIC and mass spectra were analyzed by Q-TOF. The compounds presumably produced by incubation with S9 fractions supplemented with NADP were as follows: (**a**–**c**) represent the EIC of hydroxylation and methylation (EIC 433.22000), desaturation (EIC 401.19000), and dihydroxylation (EIC 435.20000), respectively, at 240 min after incubation with swine hepatic S9. (**d**–**f**) represent their mass spectra.

The elimination of CTVD as well as the metabolites produced when CTVD was incubated with S9 fraction supplemented with NADP was investigated. NADP is a coenzyme of dehydrogenase that is often used in metabolism assays with S9 [22,23]. Incubation was conducted according to the method of Wu et al. [24]. As a result, hydroxylation and methylation, desaturation, and dihydroxylation derivatives were detected as the main metabolites of CTVD. The extracted ion chromatogram (EIC) and accurate mass spectra of these metabolites are shown in Figure A1 (Appendix A). These detected metabolites were confirmed from monoisotopic mass and mass accuracy (Table A1). Although the concentration of CTVD incubated with human hepatic S9 fraction decreased as the duration of incubation increased, the concentration of CTVD incubated with swine hepatic S9 fraction was almost unchanged from 30 min of incubation to 240 min of incubation (Figure 3a). Furthermore, the concentration of CTVD after 240 min of incubation with human hepatic S9 fraction was significantly lower than that after incubation with swine hepatic S9.

Since the hydroxide of CTVD did not show a quantitative change over time from the metabolic reaction of S9, products other than those derived from the control group due to the metabolic reaction with S9 were considered to be metabolites of CTVD. The main metabolite forms of CTVD were found to have been produced by hydroxylation and methylation, desaturation, and dihydroxylation. The main metabolites could not be quantified because standard substances of the metabolites detected were not commercially available. Thus, the metabolites in humans and swine at each time-point were compared based on the mean area of each metabolite. The metabolite peak area after incubation with human hepatic S9 fraction was two to three times higher than that after incubation with the swine hepatic S9 fraction for all metabolites (Figure 3b–d). These results indicate that human hepatic S9 fraction has a greater ability to metabolize CTVD than swine hepatic S9 fraction.

### 2.4. CTVD Elimination and the CTVD Glucuronide Profile Following Incubation with S9 In Vitro

**Figure 4:**
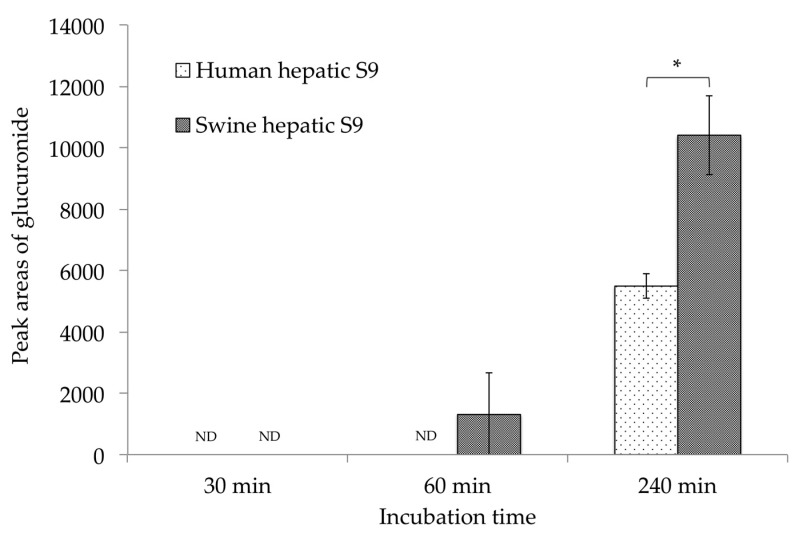
A comparison of the CTVD glucuronidation during incubation with human and swine S9 fractions supplemented with UDPGA. CTVD (1.5 µg/mL) was incubated with human and swine hepatic S9 fractions supplemented with UDPGA as a coenzyme. The amount of CTVD glucuronide at 30, 60, and 240 min was measured by Q-TOF. The metabolites in humans and swine at each time-point were compared based on the mean area of each metabolite. Values are presented as the mean ± SD. Asterisks indicate a statistically significant difference (*p* < 0.05). ND, not detected.

**Figure A2:**
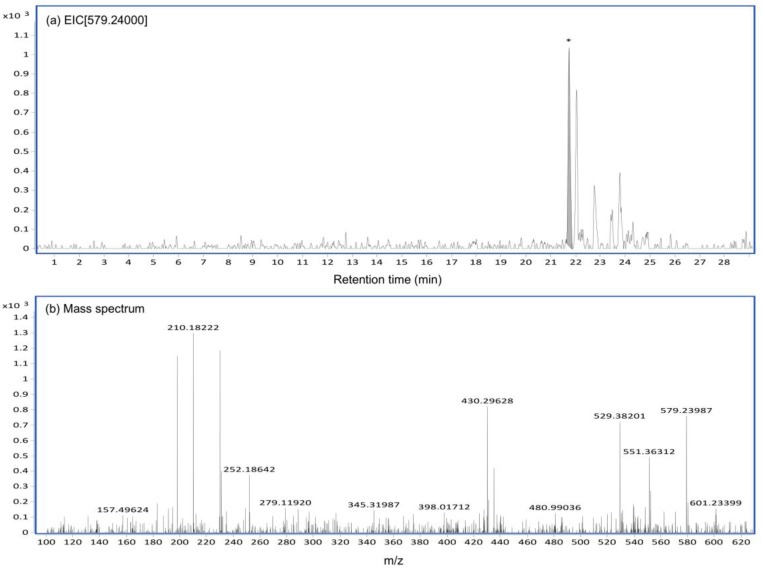
The EIC and mass spectrum of CTVD glucuronide generated by incubation with S9 including UDPGA. The EIC and mass spectrum of CTVD glucuronide produced by incubation with swine hepatic S9 supplemented with UDPGA were analyzed by Q-TOF. The EIC (**a**) and mass spectrum (**b**) of CTVD glucuronide as observed at 240 min after incubation with swine hepatic S9.

Glucuronide is a well-known metabolite produced from the detoxification of mycotoxins. However, after incubation with S9 fraction and NADP as a coenzyme, no glucuronide was recognized in our swine or human metabolite models. Moreover, it is reported that swine have low sulfate conjugation ability; this has been reported to be alternated by processes such as glucuronidation [25]. For this reason, the glucuronidation of CTVD in the presence of uridine-5′-diphosphoglucuronic acid trisodium salt (UDPGA) was examined in order to compare the detoxification ability between swine and humans. UDPGA is a cosubstrate used in the glucuronidation reaction [26]. Swine and human hepatic S9 were used for incubation, and CTVD glucuronide was observed at 30, 60, and 240 min after incubation. The EIC and accurate mass spectrum of CTVD glucuronide are shown in Figure A2. Of note, with swine hepatic S9, CTVD glucuronide was detected at 60 min and the amount increased over time until 240 min, while CTVD glucuronide was not detected at 30, 60, or 240 min with human hepatic S9 (Figure 4).

Because the standard substance of CTVD glucuronide was not commercially available, the same method that was used for the comparison of S9 fractions supplemented with NADP was applied. The mean area of CTVD glucuronide after incubation with swine hepatic S9 fraction for 240 min was approximately twice that after incubation with human hepatic S9 fraction for 240 min (Figure 4).

## 3. Discussion

Swine are known to share some physiological and anatomic similarities with humans, including food habits. The toxicokinetics of CTVD were therefore investigated in swine for extrapolation to humans. The bioavailability of mycotoxins has been reported for several major compounds. Deoxynivalenol (48–109.8% in swine) [27,28,29] and ochratoxin A (65.7% in swine) [30] have relatively high bioavailability, and the bioavailability of zearalenone in swine is reported to be 80–85% [31]. The result of the present study was over 100% by calculated from AUC∞. This was possible to have been overestimated, because the extrapolation estimate (from 48 h to infinity) in PO administration had 68% of AUC_t_. However, the bioavailability estimated from AUC_t_ (from 0 to 48 h) was 79.3%, then it was suggested that CTVD was also a mycotoxin with similarly high bioavailability to these compounds (Table 1). Generally, lipophilic substances appear to be easily absorbed by the intestine through passive transport. Aflatoxin and zearalenone are lipophilic and low-molecular mass molecules that are said to be transported through passive diffusion [32]. CTVD is a similarly lipophilic and low-molecular mycotoxin with a low-molecular mass; thus, it is considered to be absorbed by passive transport. Lipophilic compounds access the systemic circulation through the intestinal lymphatic system by which these compounds avoid the first pass effect [33]. Thus, these properties of CTVD may be one reason for its high bioavailability.

In the permeability experiment using Caco-2 cells, CTVD showed a high Papp (Table 2) despite having no marked effect on the TEER. The Papp of CTVD was similar to that of propranolol as a model of lipophilic drug [34]. Furthermore, the Papp was higher than the Papp values of deoxynivalenol (0.19 ± 0.02 [× 10^−6^ cm/min]) [35] and zearalenone (10.4 ± 4.7 [× 10^−6^ cm/s]) [36], although it was lower than that of aflatoxin M1 (105.10 ± 7.98 [×10^−6^ cm/s]) [37]. The permeability coefficient from the Caco-2 cell assay has been shown to be correlated with the bioavailability and intestinal absorbency, following a sigmoidal curve (wherein a substance with a high permeability coefficient has high absorbency) [18,19]. Considering that the results of our in vivo study using swine indicated the relatively higher bioavailability of CTVD, the result of the Caco-2 study suggested that the bioavailability of CTVD in humans would be similarly high to that in swine.

The plasma CTVD concentration showed almost no increase until 3 h after PO to swine (Figure 2b). The dwell time of digesta in the stomach of swine has been reported to range from one to three hours [38]. As CTVD was administered with feed in this study, CTVD may have been retained with digesta in the stomach. Some mycotoxins have been reported to be absorbed from the stomach [39,40]. However, in the present study, a marked increase in the plasma CTVD concentration was noted from three hours after its administration; thus, CTVD may be poorly absorbed from the stomach.

In the in vivo study, the elimination of CTVD from the body of swine appeared to be quite slow, the Vd of CTVD in swine was greater than 1 L (Table 1). Generally, drugs with a Vd exceeding 1 L are considered to be widely distributed to the body tissue [41]. Ueno et al. [12] performed an in vivo study on the distribution and elimination of CTVD, which supported our results. In their study [12], they noted that after the SC of extracted CTVD, CTVD was rapidly distributed from the site of administration to the main organs, including the liver, kidney, and heart. They also found that the concentration of CTVD was highest in the liver after 8 h, and that <1% of the total administered dose could still be detected in the liver, even after 52 h. Thus, from the results of the present study, CTVD was suggested to be widely distributed to the body tissue. Regarding other mycotoxins, ochratoxin A (84.5 h in swine, 840 h in monkey) [29,42] and aflatoxin B1 (91.8 h in rat) [43] are reported to have long elimination half-lives. In contrast, the elimination half-lives of fumonisin B1 (182 min in swine) and deoxynivalenol (7.2–15.2 h in swine) are reported to be short [44]. In this respect, CTVD is a mycotoxin with a relatively long half-life (Table 1). Hou et al. proposed that CTVD bound plasma albumin [45]. Ochratoxin A is generally accepted to bind plasma albumin [46,47], which may explain why CTVD showed a long elimination half-life. In addition, Sakai et al. reported that extract from yellow rice had a lethal effect, even when exposed rats were given daily doses of 1/300 of LD_50_ PO for 16 months [16]. In the present study, the toxicokinetics of CTVD in swine showed that CTVD had high bioavailability and persisted in the body for a relatively long period of time. This suggested that CTVD might accumulate in the body with chronic exposure, and this result was considered to reflect the above report describing the adverse effects of chronic exposure.

To estimate the bioavailability in humans, the metabolites in human hepatic S9 fraction were examined and compared to those produced by swine S9 fraction in an in vitro experiment. Regarding the metabolites present in the S9 fraction of humans, the main metabolites—including hydroxylation and methylation, desaturation, and dihydroxylation derivatives—were the same as those in the swine S9 fraction (Figure 3). Marked differences were noted in the metabolite-producing ability of the hepatic S9 fraction; the metabolization when human S9 fraction was used was higher than that when swine S9 fraction was used. Although glucuronide was detected among the metabolites produced by the hepatic S9 fractions of both species when supplemented with UDPGA, the glucuronidation of CTVD in humans was shown to be slower in comparison to in swine (Figure 4). Interspecies differences in the hepatic glucuronidation of deoxynivalenol have been reported [48]. Moreover, swine have no sulfate conjugation ability (or lower ability in comparison to other species); thus, it has been shown that sulfatic conjugation in swine occurs via pathways other than phase II pathways [25]. Furthermore, in the metabolism of CTVD, glucuronidation might compensate for sulfatic conjugation in swine due to their low sulfate conjugation ability, and this was considered to be a factor that caused the increased production of glucuronide from CTVD in pigs in comparison to humans. On the other hand, because CTVD was more metabolized in humans than in pigs, it was suggested that in humans, CTVD may be metabolized by phase II pathways other than glucuronidation. Further investigations should be performed to test this hypothesis. Overall, the results of our in vitro study using S9 fraction suggested that the metabolism of CTVD in the human liver would be faster than that in the swine liver (Figure 3).

One limitation associated with the present study is that frequent blood drawing within the first hour after administration was performed under anesthesia, due to considerations for the animals’ welfare, and that the effect in relation to absorption and metabolism was unclear. However, the results of the present study are important in that the toxicokinetic parameters in the in vivo and in vitro experiments clearly demonstrated that CTVD can easily remain in the body. This is in line with the results from previous animal experiments. In the future, although it will be necessary to accumulate further data, this research may provide useful information for evaluating the risk associated with the administration of CTVD.

Although previous animal experiments demonstrated that CTVD remains in the body for a long time, in this study we performed metric analyses to reveal the toxicokinetics for the first time. The results indicated that CTVD has high bioavailability in swine and that it persisted in the body for a relatively long time. Thus, CTVD may bring about adverse effects due to accumulation as a result of chronic exposure. In addition, the comparison of the in vitro findings in humans and swine suggested that the bioavailability of CTVD in humans was similarly high to that in swine, although CTVD appears to be metabolized more quickly in humans than in swine.

## 4. Materials and Methods

### 4.1. Reagents

CTVD (Figure 1) (purity: 88.8%) was extracted from *P. citreonigrum* isolated by Shiratori et al. [6] with reference to the method of da Rocha et al. [49]. Regarding the approximately 12% impurities, most were hydroxides of CTVD. These hydroxides did not change quantitatively during the reaction of S9 (data not shown). The Caco-2 cell lines were provided by the Division of Pharmacognosy, Phytochemistry, and Narcotics of the National Institute of Health Sciences (Kanagawa, Japan). Human and swine hepatic S9 fractions were purchased from Sekisui XenoTech, LLC. (Kansas City, KS, USA). NADP, Glucose-6-Phosphate, Hank’s balanced salt solution (HBSS) and HEPES were purchased from Sigma-Aldrich (St. Louis, MO, USA). UDPGA was obtained from Nacalai Tesque, Inc. (Kyoto, Japan). Alamethicin was obtained from LKT Laboratories, Inc. (St. Paul, MN, USA). Inactivated fetal bovine serum was obtained from Biowest (Nuaillé, France). Dulbecco’s modified Eagle’s medium (DMEM), penicillin, and streptomycin were purchased from Invitrogen Japan (Tokyo, Japan). Nonessential amino acids were obtained from MP Bio Science (Derbyshire, UK). The Corning^TM^ BioCoat^TM^ Intestinal Epithelium Differentiation Environment Kit was purchased from Corning (NY, USA). Medetomidine hydrochloride and butorphanol tartrate were obtained from Meiji Seika Pharma Co., Ltd. (Tokyo, Japan). Midazolam was obtained from Astellas Pharma Inc. (Tokyo, Japan). Other reagents were purchased from Fujifilm Wako Pure Chemical Corporation (Osaka, Japan).

### 4.2. Administration Study

#### 4.2.1. Animals and Diets

Swine (barrows; Landrace × Large White × Duroc) were obtained from CIMCO Co., Ltd. (Tokyo, Japan). They were housed in individual cages (0.88 m wide, 1.3 m deep), with ad libitum access to water, and were fed a commercial formula feed in quantities of 1.5–2% of their body weight (BW) daily. All protocols were approved by the Animal experiment ethics committee of Azabu University (Approval number: 170829-1).

#### 4.2.2. Administration and Blood Sampling

CTVD stock solution was prepared by dissolving CTVD in acetonitrile to a concentration of 10 mg/mL. The required amount of CTVD was moved from the stock solution into a tube and dried with nitrogen. The dried CTVD was then redissolved in ethanol–saline (ratio, 1:4) and was used as a test solution. Following three days of acclimatization, administration studies were carried out. CTVD (0.1 mg/kg·BW) was intravenously administered to swine (*n* = 4) via the auricular vein. For PO administration (*n* = 4), 10 mg/mL of CTVD–ethanol solution and a small amount of water were added to feed (10 g), for a dose of 0.1 mg/kg·BW. This was then fashioned into a sphere and fed to the animals at the time of feeding. It was visually confirmed that the animals had eaten the CTVD-contaminated feed.

Blood was sampled from the jugular vein at 0 min (before administration), and 5, 10, 20, and 30 min and 1, 2, 3, 4, 8, 24, and 48 h after administration. Blood samples were placed into heparinized tubes and stored on ice until centrifugation. After centrifugation (1919× *g*, 10 min), the plasma was temporarily stored at −80 °C. Plasma samples were prepared according to the method of Devreese et al. [50]. A three-fold volume of acetonitrile was mixed with the plasma samples, which were then centrifuged again (8500× *g*, 4 °C 10 min) after mixing with a vortex mixer for 15 s. The supernatants were transferred into amber screw-top vials and dried under nitrogen gas. The samples were stored at −30 °C until the analysis.

In order to frequently sample blood via a cervical part of swine in a short period of time (up to 1 h after administration), taking animal welfare into account, CTVD was administered intravenously and orally, followed by the immediate administration of 0.1 mg/kg of mixed anesthetics (medetomidine hydrochloride:midazolam:butorphanol tartrate = 3:2:2) via intramuscular injection. Therefore, blood samplings from 5 min to 1 h after CTDV administration were conducted under anesthesia. Each animal was awake at approximately 1 h after anesthetization, blood samplings from 2 to 48 h after CTDV administration were conducted under awakening.

#### 4.2.3. Toxicokinetic Analyses

The toxoicokinetics were analyzed using the Phoenix WinNonlin 6.4 software program (Certara, St. Louis, MO, USA). The bioavailability was determined using the following equation by calculating the AUC of the IV and PO data, which were determined with extrapolation to infinity.

$$F=\frac{{AUC\;}_{PO}/{Dose\;}_{PO}}{{AUC\;}_{IV}/{Dose\;}_{IV}}\times 100$$

where F is the bioavailability of CTVD. *AUC _PO_* or *_IV_* represents the mean area under the curve after PO or IV administration. *Dose* *_PO_* or *_IV_* represent the actual dose by PO or IV administration.

### 4.3. Permeability Study Using Caco-2 Cells

Cell culture and a permeability study were carried out by the method of Kadota et al. [21]. CTVD solutions (3 and 10 mmol/mL) were prepared by dissolving dried CTVD in DMSO. The permeability study was carried out using a Corning^TM^ BioCoat^TM^ Intestinal Epithelium Differentiation Environment Kit (Corning, NY, USA). Cell incubation and induction of differentiation were performed in accordance with the protocol of the kit. CTVD solutions were added to Enterocyte Differentiation Medium (EDM) containing 0.08% MITO + serum extender, with CTVD at concentrations of 3 and 10 µmol/L.

EDM containing CTVD was exposed to Caco-2 cells from the apical (AP) side. The TEER of the AP and BL sides was measured at 0 (before exposure), 1 and 2 h (after exposure) using a Millicell ERS device (Millipore, Molsheim, France). To determine the CTVD concentration at the AP and BL sides, transport buffer was collected from both sides. After transferring 400 µL of collected buffer (per side) to a microtube, a three-fold volume of acetonitrile was added. Samples were mixed with a vortex mixed, followed by centrifugation at 8500× *g* for 10 min at 4 °C. The supernatant was then transferred to an amber vial and dried with nitrogen gas. Samples were stored at −30 °C until an analysis by liquid chromatography tandem mass spectrometry (LC-MS/MS).

### 4.4. Production of CTVD Metabolites by Incubating with S9 Fractions

The incubation of S9 supplemented with NADP was carried out with reference to a previous report [24]. CTVD stock solution (25 µL) was transferred into a microtube and dried with nitrogen. CTVD solution (250 µg/mL) was prepared by redissolving dried CTVD with 1 mL of DMSO. CTVD additive solution (150 µg/mL) was prepared by mixing 600 µL of CTVD solution (250 µg/mL) and 400 µL of a base buffer. The total volume of the test solution was 500 µL. The final concentrations of each factor in the test solution were as follows: MgCl_2_ (5 mmol/L), Glucose-6-phosphate (5 mmol/L) and NADP (0.5 mmol/L). The concentrations of S9 and CTVD in the test solution were 0.5 mg/mL and 1.5 µg/mL, respectively. After adding CTVD, the test solution was incubated in a warm bath at 37 °C for 30, 60, or 240 min. The reaction of the test solution was terminated by adding the same amount (500 µL) of acetonitrile. Each sample was mixed in a vortex mixer at 30 s, followed by centrifugation at 6000× *g* for 10 min at 4 °C. The supernatant was transferred to an amber vial and dried with nitrogen gas. Dried samples were stored at −30 °C until the analysis.

S9 was incubated with UDPGA as follows: first, a mixture (S9 (final concentration, 0.5 mg/mL), Tris-HCl buffer (pH 7.4; final concentration, 50 mmol/L), MgCl_2_ (final concentration, 0.5 mg/mL), alamethicin (final concentration, 0.25 µg/mL) and CTVD (final concentration, 1.5 µg/mL)) was pre-incubated at 37 °C for 5 min. The total volume was then brought to 1 mL by adding UDPGA (final concentration, 3 mmol/L), and incubation was started at 37 °C. A 100 µL aliquot of the sample was collected from each mixture at 30, 60, and 240 min from the start of incubation. An equal amount of acetonitrile was then added to terminate the reaction. After centrifugation at 9000× *g* for 5 min at 4 °C, the supernatant was dried with nitrogen gas. Samples were stored until use at −30 °C.

### 4.5. Quantification of CTVD and Detection of Metabolites

Dried samples from the administration and permeability studies were redissolved in methanol for the analysis. The quantification of CTVD in samples was conducted under the following analytical conditions: LC was performed using an Agilent 1290 Infinity LC System (Agilent Technology Ltd., Santa Clara, CA, USA), and separation was performed using a ZORBAX Eclipse plus C18 (100 mm, 2.1 mm, 1.8 µm; Agilent Technology Ltd.). The mobile phases used were 5 mmol/L acetic ammonium and methanol, and the solvent composition was increased in a linear gradient from 50% organic modifier to 85% at 7 min. The flow rate was 0.25 mL/min, the column oven temperature was 40 °C, and the injection volume was kept at 2 µL (administration study) or 0.1 µL (permeability study). MS was performed using the Agilent 6470 Triple Quadrupole LC/MS system (Agilent Technology, Ltd.). The ion source was the Agilent Jet Stream (AJS) (Positive/Negative mode), and the drying gas temperature and flow rate were 250 °C and 10 L/min, respectively, while the sheath gas temperature and flow rate were 400 °C and 12 L/min, respectively. The fragmentor voltage was 140 V, and the nozzle voltage was 1000 V. MRM transition was performed at *m*/*z* = 403 > 139 (30 eV), 297 (15 eV). 

To detect metabolites by incubation using S9 supplemented with NADP, dried samples were re-dissolved in methanol. The LC system and analytical column were the same as described above. The mobile phases used were 0.1% formic acid and methanol, and the solvent composition was changed in a linear gradient from 10% methanol to 100% methanol in 30 min. The flow rate was 0.2 mL/min. The quantification of CTVD and the search for metabolites of CTVD were performed using an Agilent 6545 quadrupole time-of-flight mass spectrometer (Q-TOF) LC/MS system (Agilent Technologies, Ltd.). The drying gas temperature and fragmentor voltage were 350 °C and 120 V, respectively, and the other conditions were as described above. Screening for metabolites was based on a database of predicted metabolites (Table A2). Then, peaks from control were excepted.

To detect CTVD glucuronide by incubation of S9 with UDPGA, dried samples were redissolved in acetonitrile. MS was performed using the Agilent 6530 Q-TOF LC/MS system (Agilent Technology, Ltd.). The mobile phases used were 5 mmol/L acetic ammonium and methanol, and the solvent composition was increased in a linear gradient from 10% organic modifier to 100% at 30 min. The flow rate was 0.2 mL/min, the column temperature was 40 °C, and the injection volume was 3 µL. the ion source was the AJS (Positive mode). Other conditions were as described above.

### 4.6. Statistical Analyses

The mean and SD of each value were calculated. In incubation with S9, the CTVD concentration and metabolite generation rate in humans and swine were analyzed using Student’s *t*-test or Welch’s test. *p* values of <0.05 were considered to indicate statistical significance. All statistical analyses were performed using R version 3.5.0 (2018-04-23) (R Core Team [2018]. R: A language and environment for statistical computing. R Foundation for Statistical Computing, Vienna, Austria. available from: https://www.R-project.org/).

## Figures and Tables

**Table 1:** The toxicokinetic parameters in swine that received CTVD intravenously and orally.

	BW (kg)	Kel ×10^−1^ (h^−1^)	T_1/2_ (h)	Vd or Vd/F (L)	MRT (h)	AUC_t_ (h·ng/mL)	AUC∞ (h·ng/mL)	C_max_ (ng/mL)	T_max_ (h)	F_t_ (%)	F (%)
IV	9.4 ± 1.3	0.5 ± 0.1	16.2 ± 4.3	1.5 ± 0.2	14.6 ± 1.2	1322.2 ± 224.4	1512.9 ± 331.7	-	-	-	-
PO	10.7 ± 1.3	0.4 ± 0.2	21.4 ± 12.7	1.7 ± 0.3	19.6 ± 4.0	1048.2 ± 180.8	1761.1 ± 813.5	38.2 ± 6.7	15.0 ± 6.0	79.3	116.4

BW, body weight; C_max_, maximum plasma CTVD level; T_max_, time of maximum plasma CTVD concentration; Kel, the rate constants; T_1/2_, biological half-life of the elimination; Vd or Vd/F, apparent volume of distribution in IV or PO; MRT, mean residence time; AUCt, area under the curve from the curve 0 to the last quantifiable concentration; AUC∞, area under the curve from the curve 0 to infinity; F_t_, bioavailability calculated using mean AUC_t_ after PO and IV; F, bioavailability calculated using mean AUC∞ after PO and IV. With the exception of F_t_ and F, all values are presented as the mean ± SD.

**Table 2:** The Papp at each concentration of CTVD.

Parameter	3 µmol/L	10 µmol/L
Papp (×10^−6^ cm/s)	52.2 ± 28.3	42.6 ± 17.7

Papp, apparent permeability coefficient of Caco-2 cells treated with 3 and 10 µmol/L CTVD in an AP chamber for 2 h. Values are presented as the mean ± SD.

## Appendix A

**Table A1:** Information of mass spectrum for each metabolite.

Metabolites	Retention Time (min)	Polarity	Base Peak Ion	Mass Accuracy
				Relative Mass Error
Hydroxylation and methylation	25.10	Positive	(M + H)^+^	2.9
Desaturation	25.79	Positive	(M + H)^+^	1.1
Dihydroxylation	22.57	Positive	(M + H)^+^	3.1

**Table A2:** The database of predicted metabolites of CTVD.

Name	Chemical Formula	Exact Mass	Name	Chemical Formula	Exact Mass
(O, N, S) Methylation	C_24_H_32_O_6_	416.21989	Ethyl to Carboxylic Acid	C_22_H_26_O_8_	418.16277
1,4-Dihydropyridines to Pyridines	C_23_H_28_O_6_	400.18859	First/Second Alcohols to Aldehyde/Ketone	C_23_H_28_O_6_	400.18859
2-Ethoxyl to Acid	C_22_H_26_O_7_	402.16785	Glucuronidation + Hydrogenation	C_29_H_40_O_12_	580.25198
2 × Glucuronide Conjugation	C_35_H_46_O_18_	754.26841	Glucuronide Conjugation	C_29_H_38_O_12_	578.23633
2 × Hydrogenation	C_23_H_34_O_6_	406.23554	Glutamine Conjugation	C_28_H_38_NO_8_	516.25974
2 × Hydroxylation and Sulfation	C_23_H_30_O_14_S_2_	594.10770	Gluthation Conjugation	C_33_H_47_N_3_O_12_S	709.28804
2 × Hydroxylation	C_23_H_30_O_8_	434.19407	Gluthation Conjugation + Demethylation	C_32_H_43_N_3_O_12_S	693.25674
2 × Hydroxylation + 2 × Glucuronide	C_35_H_46_O_20_	786.25824	Gluthation Conjugation + Dihydroxylation	C_33_H_45_N_3_O_14_S	739.26222
2 × Oxidation + Glucuronidation	C_29_H_38_O_14_	610.22616	Gluthation Conjugation + Hydroxylation	C_33_H_45_N_3_O_13_S	723.26731
2 × Sulfate Conjugation	C_23_H_30_O_12_S_2_	562.11787	Gluthation Conjugation, Hydroxylation + Oxidation	C_33_H_43_N_3_O_14_S	737.24657
3 × Hydroxylation	C_23_H_30_O_9_	450.18898	Glycine Conjugation	C_25_H_33_NO_7_	459.22570
3 × Oxidation + Dehydrogenation	C_23_H_28_O_9_	448.17333	Hetero oxide reduction + Hydrogenation	C_23_H_32_O_5_	388.22497
Acetylation	C_25_H_32_O_7_	444.21480	Hydration, Hydrolysis (Internal)	C_23_H_32_O_7_	420.21480
Acetylation + Oxidation	C_25_H_32_O_8_	460.20972	Hydrogenation	C_23_H_32_O_6_	404.21989
Alcohols Dehydration	C_23_H_28_O_5_	384.19367	Hydrolysis + 2× Oxidation	C_23_H_32_O_9_	452.20463
Alkene to Epoxide	C_23_H_30_O_7_	418.19915	Hydroxylation	C_23_H_30_O_7_	418.19915
Alkenes to Dihydrodiol	C_23_H_32_O_8_	436.20972	Hydroxylation + Glucuronide	C_29_H_38_O_13_	594.23124
Aromatic Oxidation	C_24_H_32_O_8_	448.20972	Hydroxylation and Dehydration	C_23_H_28_O_6_	400.18859
Aromatic Ring to Arene Oxide	C_23_H_30_O_7_	418.19915	Hydroxylation and Desaturation	C_23_H_28_O_7_	416.18350
Carboxylation + Glucuronidation	C_29_H_36_O_14_	608.21051	Hydroxylation and Ketone Formation	C_23_H_28_O_8_	432.17842
Cysteine Conjugation	C_26_H_37_NO_8_S	523.22399	Hydroxylation and Methylation	C_24_H_32_O_7_	432.21480
Cysteine Conjugation and Desaturation	C_26_H_35_NO_8_S	521.20834	Hydroxylation and Sulfation	C_23_H_30_O_10_S	498.15597
Cysteine Glycine Conjugation	C_28_H_40_N_2_O_9_S	580.24545	Hydroxymethylene Loss	C_22_H_28_O_5_	372.19367
Cysteine Glycine Conjugation and Desaturation	C_28_H_38_N_2_O_9_S	578.22980	Isopropyl Dealkylation	C_20_H_24_O_6_	360.15729
Deacetylation	C_21_H_28_O_5_	360.19367	Isopropyl to Acid	C_21_H_24_O_8_	404.14712
Dacetylation + Dehydrogenation	C_21_H_26_O_5_	358.17802	Isopropyl to Alcohol	C_20_H_24_O_7_	376.15220
Debenzylation	C_16_H_24_O_6_	312.15729	Ketone to Alcohol	C_23_H_32_O_6_	404.21989
Debutylation + Hydrogenation	C_19_H_24_O_6_	348.15729	Methyl Ketone to Acid	C_21_H_26_O_7_	390.16785
Decarbonylation	C_22_H_30_O_5_	374.20932	Methylene to Ketone	C_23_H_28_O_7_	416.18350
Decarboxylation	C_22_H_30_O_4_	358.21441	N-Acethylcysteine Conjugation	C_28_H_39_NO_9_S	565.23455
Decarboxylation and Glucuronidation	C_28_H_38_O_11_	550.24141	N-Acethylcysteine Conjugation and Desaturation	C_28_H_37_NO_9_S	563.21890
Deethylation	C_21_H_26_O_6_	374.17294	Oxidation + 2× Desaturation	C_23_H_26_O_7_	414.16785
Demethylation	C_22_H_28_O_6_	388.18859	Oxidation + Acethylcysteination	C_28_H_39_NO_10_S	581.22947
Demethylation + Dehydrogenation	C_22_H_26_O_6_	386.17294	Oxidation + Deacetylation	C_21_H_28_O_6_	376.18859
Demethylation + Glucuronidation	C_28_H_36_O_12_	564.22068	Oxidation + Demethylation + Dehydrogenation	C_22_H_26_O_7_	402.16785
Demethylation + Hydrogenation	C_22_H_30_O_6_	390.20424	Parent	C_23_H_30_O_6_	402.20424
Demethylation + Oxidation + Glucuronidation	C_28_H_36_O_13_	580.21559	Phosphorylation	C_23_H_31_O_9_P	482.17057
Demethylation and Hydroxylation	C_22_H_28_O_7_	404.18350	Propyl Ether to Acid	C_20_H_22_O_7_	374.13655
Demethylation and Methylene to Ketone	C_22_H_26_O_7_	402.16785	Propyl Ketone to Acid	C_19_H_22_O_7_	362.13655
Demethylation and two Hydroxylations	C_22_H_28_O_8_	420.17842	Quinone Formation	C_23_H_28_O_8_	432.17842
Demethylation to Carboxylic Acid	C_23_H_28_O_8_	432.17842	Sulfate Conjugation	C_23_H_30_O_9_S	482.16105
Desaturation	C_23_H_28_O_6_	400.18859	Taurine Conjugation	C_25_H_35_NO_8_S	509.20834
Desaturation + Gluthation Conjugation	C_33_H_45_N_3_O_12_S	707.27239	Tert-Butyl Dealkylation	C_19_H_22_O_6_	346.14164
Epoxidation + Gluthation Conjugation	C_33_H_47_N_3_O_13_S	725.28296	Tert-Butyl to Acid	C_20_H_22_O_8_	390.13147
Ethyl Ether to Acid	C_21_H_24_O_7_	388.15220	Tert-Butyl to Alcohol	C_19_H_22_O_7_	362.13655
Ethyl Ketone to Acid	C_20_H_24_O_7_	376.15220	Triphosphorylation	C_23_H_33_O_15_P_3_	642.10323
Ethyl to Alcohol	C_21_H_26_O_7_	390.16785	Two Sequential Desaturations	C_23_H_26_O_6_	398.17294
